# Roads Forward for European GMO Policy—Uncertainties in Wake of ECJ Judgment Have to be Mitigated by Regulatory Reform

**DOI:** 10.3389/fbioe.2019.00132

**Published:** 2019-06-05

**Authors:** Martin Wasmer

**Affiliations:** Centre for Ethics and Law in the Life Sciences (CELLS), Leibniz University Hannover, Hanover, Germany

**Keywords:** GMO regulation, future policy, CJEU C-528/16, directive 2001/18/EC, genome editing, new genetic modification techniques (nGM), CRISPR/Cas, directed mutagenesis

## Abstract

This article gives an overview of legal and procedural uncertainties regarding genome edited organisms and possible ways forward for European GMO policy. After a recent judgment by the European Court of Justice (ECJ judgment of 25 July 2018, C-528/16), organisms obtained by techniques of genome editing are GMOs and subject to the same obligations as transgenic organisms. Uncertainties emerge if genome edited organisms cannot be distinguished from organisms bred by conventional techniques, such as crossing or random mutagenesis. In this case, identical organisms can be subject to either GMO law or exempt from regulation because of the use of a technique that cannot be identified. Regulatory agencies might not be able to enforce GMO law for such cases in the long term. As other jurisdictions do not regulate such organisms as GMOs, accidental imports might occur and undermine European GMO regulation. In the near future, the EU Commission as well as European and national regulatory agencies will decide on how to apply the updated interpretation of the law. In order to mitigate current legal and procedural uncertainties, a first step forward lies in updating all guidance documents to specifically address genome editing specifically address genome editing, including a solution for providing a unique identifier. In part, the authorization procedure for GMO release can be tailored to different types of organisms by making use of existing flexibilities in GMO law. However, only an amendment to the regulations that govern the process of authorization for GMO release can substantially lower the burden for innovators. In a second step, any way forward has to aim at amending, supplementing or replacing the European GMO Directive (2001/18/EC). The policy options presented in this article presuppose political readiness for reform. This may not be realistic in the current political situation. However, if the problems of current GMO law are just ignored, European competitiveness and research in green biotechnology will suffer.

## Introduction

This article gives a brief overview (section The ECJ Judgment and Its Ramifications) of what kinds of problems the European Union's (EU) regulatory framework for genetically modified organisms (GMOs) faces in the wake of the ECJ judgment *Confédération paysanne a.o*. on directed mutagenesis (ECJ, 2018). In a second step (section Roads Forward), policy options are discussed that could avert a crisis for European agricultural innovators and a crisis of enforcement for regulatory agencies. This crisis results from the inadequacy of the current regulatory framework to proportionately, predictably, and enforceably regulate organisms that have been bred by genome editing.

The term genome editing as used in this article refers to a variety of new techniques, specifically techniques of directed mutagenesis using CRISPR/Cas9 (or similar site-directed enzymatic DNA cleavage or base-editing in the sense of SDN1/2; see Podevin et al., 2013), or other techniques such as oligonucleotide directed mutagenesis. These techniques allow breeding organisms in which the genetic material has been altered to an outcome that is genetically indistinguishable from the possible outcomes of conventional breeding, i.e., traditional breeding by crossing and natural variation as well as conventionally used techniques of chemically or radiation-induced random mutagenesis. Throughout this article, the term “genome edited organism” (GEO) refers to an organism that has been altered by such techniques to an outcome that cannot be distinguished from a conventionally bred variety or a naturally occurring variant thereof. Note that this use of the term does not include every alteration that is possible with these same techniques; especially it excludes transgenic modifications (e.g., SDN3 with CRISPR/Cas9 and donor DNA with a sequence from another species as repair template; see Podevin et al., 2013). That is to say, the definition of GEO used throughout this article is outcome based, not process based. The same techniques can also be employed to alter an organism in a way that is easily distinguishable from conventionally bred varieties or a naturally occurring variant. The focus of this article, however, is on organisms that cannot be distinguished.

A reference scenario for the cases discussed in the following is a GEO that contains a point mutation, which provokes a frameshift in the DNA code or changes the code to form a stop-codon, both of which may knock-out a certain gene (loss of function mutation). For example, resistance to powdery mildew in barley occurs naturally and is caused by a loss of function mutation in the *Mlo* gene. Genome editing can place a point mutation in the equivalent of that gene in barley varieties that are not mildew resistant yet or in other plants that are susceptible to mildew such as wheat or tomato (e.g., Acevedo-Garcia et al., 2017; Nekrasov et al., 2017). A point mutation, such as a frameshift mutation in the *Mlo* gene in a barley variety, can be detected by sequencing if the sequence of the parent organisms is known. But it is often not possible to identify the cause of the mutation, i.e., establish whether the mutation occurred naturally or by conventional random mutagenesis and subsequent backcrossing or through directed mutagenesis (see e.g., Lusser et al., 2012; Bartsch et al., 2018; Grohmann et al., 2019). Indeed, if the only alteration introduced is nothing else than one single base mutation, then it is not at all possible to identify the technique used. The reason for this is that a wide variety of mutations in the genome occurs constantly in nature, most often during reproduction. In particular, double strand breaks of the DNA occur naturally and are subsequently repaired by the natural cellular repair mechanisms of non-homologous end joining and homology-directed repair. Those are the same kind of breaks in the DNA that can be introduced by the CRISPR associated enzyme CAS9 (and others). And the subsequent cellular repair mechanisms (non-homologous end joining and homology directed repair) are also the same as used in directed mutagenesis used in directed mutagenesis. In addition, a fabricated nucleic acids template (donor DNA) may be introduced in the lab to control the outcome of the mutation event and, e.g., accurately reproduce an alteration that is known to have emerged naturally in that variety. In consequence, for some small alterations that blend well into the genetic background (such as a single nucleotide frameshift mutation) it is impossible (without additional knowledge such as e.g., lab reports) to identify whether they occurred naturally or whether they are human made. Such identification based on sequence data alone is indeed even impossible on theoretical grounds, unless a technique were to preferentially incorporate certain isotopes or leave an epigenetic pattern, which however is not currently known and most certainly might only apply to single techniques under very specific conditions. It is true that if more genes or more copies of a gene (e.g., in polyploid organisms) have been altered in the same fashion, probabilistic considerations could provide evidence for the use of genome editing or similar techniques. However, data on intraspecies variation (for specific varieties and even for specific loci) is quite sparse for most plant species and even for many agriculturally relevant crops (e.g., see Jiao et al., 2012 for an assessment of genetic changes in conventional maize breeding and note how sparse the data seems to be for this major crop). The lack of knowledge on natural occurrences of mutations makes it difficult to establish whether a mutation is reasonably possible to occur naturally or not (a brief overview on comparison with naturally occurring mutations is given by Custers et al., 2019). Thus, on sequence data alone, a small alteration as discussed in this scenario is only detectable if a comparator sequence is given. If no relevant information is given in addition to the sequence—e.g., when controlling imports of agricultural commodities–small alterations made by genome editing can often neither be detected (because of the reasons listed above) nor is the technique identifiable that led to the alterations. In particular, this would be the case in our reference scenario of *Mlo* locus altered barley.

For further discussion on existing and upcoming detection and identification strategies, see Grohmann et al. (2019).

## The ECJ Judgment and its Ramifications

On July 25th 2018, the European Court of Justice delivered its judgment in a case concerning among others the scope of the mutagenesis exemption in the European GMO Directive (ECJ, 2018). For a detailed analysis of the judgment see e.g., Seitz (2018) or Faltus (2018), a brief analysis in English is given by Purnhagen et al. (2018b) and Garnett and Beck (2018). The ruling has the following implications:

1. The GMO definition in Art. 2(2) i.c.w. Annex I A of the GMO Directive 2001/18/EC^1^, taking into account the reference to techniques of genetic modification in Art. 3 i.c.w. Annex I B, applies to all organisms in which the genetic material has been altered by techniques of mutagenesis (ECJ, 2018, 32-38). This means that it does not matter what the alterations to the genetic material of an organism are and whether they are extensive or insignificantly small, any organism bred with a technique of (random or directed) mutagenesis is in legal terms a GMO.
2. The exemption for organisms produced by mutagenesis from the obligations of the GMO Directive (mutagenesis exemption in Dir. 2001/18/EC, Art. 3 i.c.w. Annex I B) applies only to techniques of mutagenesis that “have conventionally been used in a number of applications and have a long safety record” (the Court here refers to Rec. 17: ECJ, 2018, para 44-46, 48-53). Referring to the precautionary principle (para 47-50), the Court further interprets the aforementioned wording of Rec. 17 as not applying to techniques “which have appeared or have been mostly developed since the Directive was adopted” (ECJ, 2018, para 51). Thus, techniques of genome editing—which have been mostly developed after adoption of the Directive in the year 2001—are not exempted from the obligations of GMO law.

In consequence, the ECJ does not differentiate between GEOs and transgenic organisms in all respects. Both are regulated as GMOs and subject to the same obligations, i.e., risk assessment, expiring market approval, post-release monitoring, liability, labeling. Indeed, the Court's reading of the Directive's GMO definition and mutagenesis exemption also applies to “downstream” directives and regulations that interact with the GMO definition i.c.w. Annex IB of the GMO Directive (ECJ, 2018, para 60-68; Purnhagen et al., 2018b).

This verdict has left scientists, breeders as well as officials from regulatory agencies perplex (the competent authorities of several countries, among them Sweden and Germany, assumed a differential treatment of GEOs before the ECJ judgment, e.g., see BVL, 2017; Eriksson, 2018a). The ruling ultimately reflects the fundamental problem of European GMO law: Long before the request for a preliminary ruling had been addressed to the ECJ, the legislator failed to acknowledge and incorporate decades of technologic development, especially the capacity of new techniques to alter the genetic material of organisms to a result that is indistinguishable from conventional breeding or natural variation.

Three major uncertainties result from the failure to account for technological change:

First and foremost, regulatory agencies will have a hard time implementing the verdict because they do not have the means to enforce compliance with GMO legislation in the case of fraudulent or unintentional non-declaration (cf. Faltus, 2018). Several large exporting nations of agricultural products outside of the EU have chosen to regulate (at least some) GEOs no different from conventionally bred varieties, thus not requiring any tracing or labeling of those GEOs (cf. Sprink et al., 2016; BMEL, 2018; Duensing et al., 2018; Wolt and Wolf, 2018). Non-declaration is then particularly likely to occur in international trade with agricultural commodities and it is precisely where regulatory agencies will fail for a variety of reasons. GEOs are in practice not distinguishable from conventionally bred varieties (discussion above). Since the technique is regulated as conventional breeding in several non-EU jurisdictions, soon a large number of varieties will be brought to market outside of the EU, without any notification procedures in most countries. European regulatory agencies would now have to somehow keep track of all of them, in order to identify them. This affects the enforcement of the regulations on deliberate release of GMOs (as generally laid down in the Directive 2001/18/EC), the enforcement of the regulations on (unintentional) transboundary movements of GMOs (as laid down in Regulation EC No 1946/2003^2^), the ability of authorities to enforce the compliance with traceability regulations (as laid down in Regulation EC No 1829/2003^3^ and No 1830/2003), the ability of authorities to enforce the 0.9% tolerance threshold for conventional products contaminated by GMOs (as laid down in Regulation EC No 1830/2003^4^) and finally the ability of authorities to enforce the EU's zero tolerance policy for unauthorized GMOs, particularly in the case of agricultural commodities. For such regulatory enforcement, there are hardly enough inspectors and technical means (such as next-generation whole genome sequencing machines) as well as not adequate means for investigation with which to retrace complex malpractices if only isolated accidental mis-declaration of agricultural goods is given as probable cause. In fact, cases of unauthorized release that have only been noticed after years of malpractice exist even with conventional transgenic GMOs such as in the petunia case (Bashandy and Teeri, 2017), despite the fact that transgenic organisms should be comparably easy to identify. In addition, sooner or later a few rouge breeders that release GEOs in their gardens or fields might come to public attention, similarly to the case of “CRISPR cabbage,” where the involved plant geneticist mocked authorities by stating “if I don't tell you, [which alterations I made to the cabbage planted in my garden] you will not find out” (Kupferschmidt, 2018). Public interest in such cases will not cease while regulatory agencies remain unable to enforce the ECJ judgment outside of medium to large-sized breeding companies that apply for commercial permits and patents for their new breeds. Such a situation can erode confidence in Union legislation and in the capability of authorities to keep food and feed products on the European market safe.

Second, uncertainties arise regarding the specifics in the procedure of approval of GMOs (for a brief overview of the assessment procedure and its challenges, see Halford, 2019; Schiemann et al., 2019). Currently passing an application for placing on the market of a GMO (especially for cultivation and in some member states even for field trials) represents a significant regulatory barrier for innovators. The big driver of cost of such an application is its long and unpredictable duration, as a notifier might be asked to provide additional evidence midway through the process of application for authorization of GMO release. Compared to conventional GMOs however, the uncertainties for innovators that want to bring GEOs to market are far superior, because is yet unknown how applications of all kinds will be handled in the case of organisms that resemble conventionally bred varieties (i.e., for field trials, for market-release of non-food&feed and for food&feed). For example, when applying for market-release, notifiers must provide detection and identification techniques (unique identifier) in order to reliably distinguish the GMO in question from any other organisms (Directive 2001/18/EC, Annex III A, sec. II, C, 2(f) and Annex III B, sec. I & II, B, 5, as amended by Commission Directive 2018/350)^5^. Will the regulatory agencies just accept a reference to the specific DNA-sequence of the alteration? Or will they refuse some GEOs on the account that the alteration is too small to allow for reliable identification? Or will they even resort to asking breeders to incorporate a transgenic “marker sequence” to facilitate tracing of GEOs? Similarly, it is not clear yet which methods of identification will be accepted with regard to the environmental risk assessment that is intended to ascertain among others whether the alteration of a GMO is not transferred to the environment (especially if the GEO in question is not necessarily distinguishable from organisms present at the site of release). How regulatory agencies will implement the judgment regarding such issues is yet unknown and most companies will stop product development for the European market under these uncertain conditions.

Third, wide-ranging uncertainties remain concerning the interpretation and the legal effects of the ECJ judgment.

- How safe is safe enough? The ECJ, following the referring court, qualified techniques of directed mutagenesis as techniques of which “the risks for the environment or for human health have not thus far been established with certainty” (ECJ, 2018, para 47). Based mainly on precautionary considerations and on the wording of Recital 17 (which requires a “long safety record”), the Court judged that these techniques are not excluded from GMO law by the mutagenesis exemption. However, knowledge on the safety of these technologies will change over time, as does the length of their safety record. This raises legal uncertainties as to what happens when—at some point in the future—the technology of genome editing can be considered safe with certainty (e.g., if in 20 years genome editing is routinely used for medical applications). Will the judgment have to be interpreted differently then?
- Which technologies exactly are excluded from the obligations of the Directive? The court held that the mutagenesis exemption does not apply to “techniques/methods of mutagenesis which have appeared or have been mostly developed since adoption of the Directive,” that is to say since the year 2001 (ECJ, 2018, para 51). Finding out which technologies are meant requires a thorough historical study of breeding techniques in that time. Note that the history is quite intricate. Some techniques of directed mutagenesis have appeared well before the year 2001 and had even some history of development and use in plants, albeit not widespread commercial use (e.g., oligonucleotide-directed mutagenesis in maize or tobacco, see Beetham et al., 1999; Zhu et al., 1999). On the other hand, some techniques of chemically or radiation induced random mutagenesis in use today can be considered to have been mostly developed after the year 2001, even more so regarding commercial applications in plant variety breeding (e.g., ion-beam mutagenesis, see Matsumura et al., 2010). In fact, the methods and techniques used in “conventional” mutation breeding do also progress rapidly and markers, dosages as well as mutagens used today significantly differ from the ones used at the time of adoption of the Directive–technological progress did not stop (a brief overview is given by Oladosu et al., 2016). In short, which technologies exactly are excluded by the judgment (ECJ, 2018, para 51) is not evident. But did the Court really make the interpretation of the mutagenesis exemption dependent on the details of a historical study of mutagenesis breeding techniques? If not, then the judgment must somehow explain a categorical distinction that underlies the historical argument. Indeed, in the buildup of the interpretation of Rec. 17 (ECJ, 2018, para 45-51) the ECJ assigns different categories of risk to all random mutagenesis techniques on the one hand and all techniques of directed mutagenesis on the other. Making reference to the findings of the referring court, the ECJ regards the techniques of directed mutagenesis (in bulk) as a risk on account of their ability “to produce […] varieties at a rate and in quantities quite unlike those resulting from the application of conventional methods of random mutagenesis” (ECJ, 2018, para 48). Thereby it clearly does not hold a historical argument applying to all newer mutagenesis techniques equally, but it differentiates between random techniques of mutagenesis on one side and directed mutagenesis on the other. The Court however did not further clarify wherein the risk consists in such a case. Does the Court deem the speed of the breeding process as a risk in itself or the ease of application of new techniques of directed mutagenesis? At least one of the two seems to be the case and this argument seems to be more important to the court than the historical details of the technological development. However, all new breeding technologies could be a risk in itself in this sense. How then will different kinds of future technologies be valuated, if they should allow an improved efficiency in breeding? Agreement on which techniques of mutagenesis are excluded from the obligations of GMO law after the ECJ judgment and which not is hardly possible unless the ECJs criteria are clarified. This is not least the case for new random mutagenesis techniques (i.e., not directed mutagenesis) that have been mostly developed after adoption of the Directive (and concomitantly do/did not have a long safety record).
- How far-reaching are the implications of the judgment? It is clear that the judgment also impacts directives and regulations “downstream” of the GMO Directive (i.e., regulations that depend directly or indirectly on and/or are affected by the mutagenesis exemption of the Directive). For instance, the ECJ was clear that the judgment is also relevant to the common catalog of varieties of Directive 2002/53 (ECJ, 2018, para 58-60). But do some of these considerations also apply to the Directive 2009/41/EC^6^ on the contained use of GM micro-organisms (cf. Kahrmann and Leggewie, 2018)? Either way, some member states might have to revise their own national GMO laws and regulations as a consequence of the judgment, as their wording does not conform to the new interpretation of the mutagenesis exemption. Furthermore, the judgment raised a methodological question regarding ECJ case law. Did the Court mean to set a precedent and (generally or under specific circumstances) revert to a historic reading instead of a dynamic interpretation of undefined legal terms? If that were the case, the judgment would have a huge and lasting effect on biotechnology law (Seitz, 2018).

Only further clarifications by the ECJ can finally settle these legal uncertainties. Given current uncertainties, it is possible that within the next years another national court will refer similar or entirely different questions regarding Directive 2001/18/EC in a preliminary ruling procedure, that have an effect on the interpretation of the judgment. In fact, concerned individuals or organizations who have the time, risk-readiness and funds might take the initiative and initiate a law suit to probe the ECJs judgment (see corresponding remarks in Table 1). In addition, a national court would have to be willing to draft appropriate questions. On the other hand, there is the option of legal change.

**Table 1:** Table of genome editing directed policy options for different actors. Sorted by timing.

**Action**	**Actors**	**Start of effect^a^**	**Effect**
Make use of flexibility within current legal framework: – Transparency: revise guidance documents explicitly addressing GEOs – Predictability: if not scientifically warranted, do not ask for additional information or further assessments – Lower barriers for GEO authorization, especially for field trials and non-food&feed release	Regulatory agencies in member states and on European level	1 year	(Slightly) lowering legal uncertainty
Amend Commission Implementing Regulation (EU) No 503/2013: – Scale process of approval to allow differential treatment for different types of gmos, i.e., explicitly addressing geos – Relax ERA duration and number of locations, adapt to hypothesis of risk – 90 days feed studies only where hypothesis of risk – Relax monitoring modalities to hypothesis of risk	EU commission	2-5 years	Lowering cost for innovators
Update Annexes II, III, VI, VII of GMO Directive 2001/18/EC: – Scale process of approval to allow differential treatment for different types of GMOs, i.e., explicitly addressing GEOs	EU commission	2-5 years	Lowering cost for innovators
Probe ECJ judgment by putting new cases to trial: – Clarify what is a long safety record for mutagenesis – Clarify if every smallest base edit really leads to GMO in the sense of Dir. 2001/18/EC Art. 2(2) – Clarify how judgment is enforced regarding non-detectability	Various stakeholders	3-10+ years	Lowering legal uncertainty
Amend, supplement or replace GMO law (primarily Dir. 2001/18/EC): – Amend Dir. 2001/18/EC Art. 2(2) or Art. 3 i.c.w. Annex IB to exclude geos – Amend Dir. 2001/18/EC Rec. 17: What is long safety record? – Supplement by specific act for geos – Replace by outcome-based regulation (e.g., novel trait) – Replace by sectorial law	EU parliament & commission	5-10+ years	Solves all issues, including enforcement issue
Develop new technologies (speculative): – Alternatives to altering germline DNA for breeding e.g., epigenetic modifications, mRNA interference or proteome modifications are not currently regulated – Alternative employments of techniques of conventional (by chemicals or radiation, *in vivo*) mutagenesis	Basic research	Decades	Circumventing regulation

^1^*Commission Directive EU No 2018/350 of 8 March 2018 amending Directive 2001/18/EC of the European Parliament and of the Council as Regards the Environmental Risk Assessment of Genetically Modified Organisms. 67, 30-45. Available online at: http://data.europa.eu/eli/dir/2018/350/oj (accessed September 12, 2018)*.

a * All timespans given in Table are only rough estimates. The values for “start of effect” are estimated based on the following evidence: The duration of release or amendment of regulations by the parliament and/or by the commission can be estimated based on the history of e.g, Regulation (EC) No 1829/2003 and Commission Regulation (EC) No 641/2004. Regulation (EC) No 1829/2003 was adopted by the commission on 25.7.2001 and the date of its official publication was 22.9.2003, i.e., 2 years (without timespan for implementation). In the case of Commission Regulation (EC) No 641/2004, which is based on Regulation (EC) No 1829/2003 the date of effect is 7.4.2004, which is presumably even less than one year after drafting. Other regulations have been released within similar timeframes. The duration of the committee procedure of article 27 i.c.w. Art. 30(2) of the GMO Directive 2001/18/EC can be estimated on prior instances of its application. On 8.3.2018 changes to various Annexes of the GMO Directive were implemented (Commission Directive EU No 2018/350) involving a committee procedure, whereby the first draft was published on 10.11.2016, giving the procedure a total timespan from drafting to publication of c. 1.5 years. The estimate was heightened to 2–5 years since changes to the annexes of Directive 2001/18/EC are only effective in combination with amendments in the corresponding regulations. The estimates of 2–5 years given above are slightly more generous, to account for the politically delicate nature of the amendments and a longer timespan required by authorities to apply new legislation in the regulatory process. Amendments to the Directive have been brought into force on four dates (07.11.2003; 21.03.2008; 02.04.2015; 29.03.2018) between publication of Directive 2001/18/EC and Nov 2018 (see history of amendment available on http://data.europa.eu/eli/dir/2001/18/oj), which allows to deduce a span of 3-8 years. The duration of a major redrafting and replacement of the entire Directive was estimated as 5-10+ years based on the timeframe it took for Directive 2001/18/EC of 12 March 2001 to be drafted and replace its antecessor Directive 90/220/EEC of 23 April 1990. The process of redrafting did of course not start immediately after publication of 90/220/EEC but only a few years later. The first proposal by the commission was published on 26 Nov 1997, hence the estimate of a minimum of 5 years. After implementation into the directive of the above legislative procedures on European level, transposition by member states might take about 1.5 years (e.g., this is the timeframe for Commission Directive EU 2018/350). The duration of legal actions that involve a clarification of GMO law is very unpredictable and the vague span of 3-10+ years reflect this fact. Finally, research and development of new breeding techniques is ongoing (e.g., CRISPR mediated epigenetic modification) but technologies developed in basic research usually take decades to be transferred to market readiness*.

Any of these three categories of uncertainties weighs heavily on research and development decisions, not only in large multinational companies but also all the way down to basic research in plant science and agricultural systems (Smyth and Lassoued, 2019; Zimny et al., 2019). It is not surprising that calls to urgent action are soaring, such as a recent call signed by scientists from 118 life sciences research institutions (VIB, 2018). If the situation remains unchanged, this will soon result in loss of competitiveness for Europe's green-biotech industry, for breeding and seed industries and for European agriculture. In addition, trade disruptions and concomitant economic consequences might follow if the zero-tolerance policy is indeed enforced and imports are halted by authorities based on suspected low-level presence of unapproved GEOs (Ryan and Smyth, 2012; cf. Kalaitzandonakes et al., 2014). Even a WTO trade dispute might ensue, similar to the *USA, Canada et al. vs. EU* cases DS291-293 on the import of GMOs to the European market, that ended in disadvantage for the EU (cf. WTO Reports of the Panel, 2006). Indeed, ten countries have already taken issue with the disruptive consequences of the ECJ verdict for international trade, demanding to “avoid arbitrary and unjustifiable distinctions between end products derived from precision biotechnology and similar end products obtained through other production methods” (WTO, 2018).

It is now the task of the Union legislator to update regulation in order to adequately reflect recent developments in breeding techniques and to prevent a crisis of enforcement.

## Roads Forward

Any way forward from the status-quo has to address present uncertainties and deliver solutions tailored to new breeding techniques. A number of possible courses of action are feasible and allow for a sustainable development of European GMO policy that has the potential to bring Europe back on track in agricultural biotechnology (see Table 1).

### Make Use of Flexibility Within Current Legal Framework

GEOs do not fit squarely into the current regulatory framework (discussion above), mainly because legally they are GMOs but biologically they often are indistinguishable from organisms bred by conventional techniques that are not regulated as GMOs. Regulatory agencies therefore need to use the flexibility they have legally in their disposition to tailor regulatory processes to GEOs.

There are two main ways of authorization of GMOs in the EU, depending on the goal of the applicant (Voss, 2006; Roïz, 2014): Authorization for the deliberate release into the environment of a GMO (according to Directive 2001/18/EC) is the “default” way of authorization. It is possible to apply for an authorization for placing on the market of a GMO for any commercial purpose, i.e., cultivation, importation or transformation of GMOs into industrial products (according to Directive 2001/18/EC, part C). Typical examples are the importation of GMO flowers or the placing on the market of MON810 seeds for cultivation. It is also possible to apply for deliberate release for non-commercial purposes, i.e., an experimental release such as a field trial (according to Directive 2001/18/EC, part B). Authorization for placing on the market of GMOs as food and feed is the way to go for all products that are intended for food or feed use and contain GMOs, are produced from GMOs or contain GMO ingredients (according to Regulation 1829/2003 and Commission Implementing Regulation EU No 503/2013). An authorization for cultivation of a GMO for food and feed production can also be obtained in this way. Obtaining authorization for the import of GMO commodities is easier than for cultivation. While MON810 is the only GMO currently authorized for cultivation in the EU (but opted-out by 19 EU member states), numerous GMOs are authorized for various uses other than cultivation (see the GMO registers at https://webgate.ec.europa.eu/dyna/gm_register/index_en.cfm and http://gmoinfo.jrc.ec.europa.eu/gmc_browse.aspx; pers. comm. by a reviewer).

To carry out these processes of authorization, multiple regulatory agencies are involved on all levels from EU to the member states regions in some cases. Member states institutions alone are responsible for authorization of field trials (national risk assessment and national authorization) but member states and EU institutions are jointly responsible for market authorization of GMOs (for import of commodities, for cultivation and as food and feed), in which case an EU-wide authorization process including risk assessment is carried out by EFSA and supported by national authorities (Directive 2001/18/EC^1^ part B and part C respectively, and Regulation 1829/2003). In addition, in the case of cultivation in a certain member state, obtaining a national risk assessment is required.

In practice, regulatory agencies are always subject to member states' or the union's political interests and–legal obligations set aside—their attitude regarding smooth and efficient administrative procedures goes a long way toward providing an innovation friendly regulatory landscape. In the next few months, all regulatory agencies (partly independently on EU level and in member states) will decide on how to implement the ECJs judgment. They have considerable freedom in doing so and their decisions will certainly at least slightly reshape the regulatory landscape—for better or for worse.

To address the aforementioned uncertainties and avoid a disproportionate regulatory burden for GEOs, the following considerations should guide regulatory agencies in implementing the ECJs judgment:

First and foremost, given the complex situation in the aftermath of the judgment, transparency is key. To reduce uncertainty regarding GEOs, it is important that regulatory agencies communicate transparently how GEOs will be treated by publishing new guidelines (e.g., EFSA guidance documents) that explicitly cover organisms gained by all relevant new techniques and different kinds of alterations. All possible obstacles in the process of application that could arise with GEOs should be addressed in guidance documents in order to (at least slightly) lowering legal uncertainty. It is the duty of regulatory agencies to ensure a high predictability of regulatory decisions and transparent and simple guidelines are a first step.

Second, the process of authorization should take account of the indistinguishability of GEOs from conventionally bred organisms. Thereby, it is the regulatory agencies duty to evaluate organisms gained by new techniques on scientific basis alone. While the ECJ has invoked the precautionary principle based on the mere possibility of risks (ECJ, 2018, para 48) as a reason to place new techniques under GMO regulation, the risk assessments in applications for authorization of GMOs within the bounds of those laws can only be based on scientific method and scientific evidence. GEOs that cannot be distinguished from conventionally bred varieties cannot involve more or less risks than these conventionally bred varieties themselves, unless the risks do not stem from the alterations made to the genetic material of the organism but instead from the methods used (for instance by introducing unintended alterations that amount to a relevant new trait but somehow escape our notice; it is a bit ironic then that the ECJ judgment, para 48, designates haphazard alterations as not presenting a risk in organisms altered by chemical and radiation-induced mutagenesis whereas it is presumably regarded as the main source of risk in GEOs). Anyhow, there is currently scientific consensus that the new techniques are in principle safe (Leopoldina, 2015; Gao et al., 2018; VIB, 2018; cf. Diekämper et al., 2018) and this consensus is successfully paving the field for medical applications of the same techniques (e.g., Baylis and McLeod, 2017; Ginn et al., 2018). Pre-release monitoring is a legal requirement and therefore at least a standard evaluation has to be conducted also on GEOs, even if no particular risks are expected to be associated with the specific alterations introduced into the organism. But it is not possible to empirically assess risks that are not known and cannot be foreseen, i.e., for which there is no scientific hypothesis to test for. When confronted with such risks, scientists have two options. A disproportionate option is to test various kinds of evidence at random and hope for a serendipitous discovery of a hazard. In such a case however, there is no scientific measure by which testing can be completed and no reason for preferring one kind of evidence over another (in simple words: how is someone to decide at which point to end testing, if nothing is found?). The only judicious option is to perform a few standard tests and focus on facilitating early detection in post-release monitoring (in simple words: if nothing relevant came up in standard testing, assume all is good. But if ever a hazard comes up, be ready and react quickly). Therefore, as long as not even a hypothesis is given as to how an organism, e.g., barley with an *Mlo* point mutation, that has been bred by genome editing has higher risks than another organism bred by “conventional” techniques, e.g., barley with the same *Mlo* point mutation, then testing should stop after a few empirically meaningful standard tests. This second option is the one that regulatory agencies should apply to GEOs, as long as no hypothesis of risk is given, neither from the technology nor from the organism's traits.

Indeed, the GMO Directive (2001/18/EC) leaves some freedom to tailor the regulatory process to cases of low risk, such as most GEOs:

First, it was one of the goals of the 2001 amendment to fixate (and thereby shorten) the duration of single steps and the overall duration of the process of application for authorization of GMO release (Voss, 2006). The timespan from application to decision could be reduced in principle to a swift 6–9 months for market-release in the case of cultivation (for an in-depth discussion of timing in the process of authorization see Voss, 2006). To keep up with the timeframe envisaged in the Directive it is however crucial that authorities do not ask for additional information after notification, as this allows prolonging the timespan beyond the norm. Consequently, the Directive stresses that authorities requesting additional information require a solid reason for so doing (cf. Directive 2001/18/EC, Art. 6(7), Art. 14(4), Art 15(1), Art 18(1)). As GEOs in most cases do not present a scientifically grounded risk (discussion above), there can be no warranted reason for an authority to prolong the process by asking for further tests or additional information, unless in response to new scientific evidence.

Second, the Directive (2001/18/EC) allows for adapting the modalities of assessment to different types of GMOs and their different concomitant risks. Its Art 7(1) allows for the application of differentiated (simplified) procedures if sufficient experience has been obtained by releases of certain GMOs in certain ecosystems and sufficient evidence of safety is available (Directive 2001/18/EC^1^ Art. 7(1) i.c.w. Annex V). For placing on the market of certain types of GMOs, a competent authority or the Commission may propose to derogate from the general requirements for the notification procedure Directive 2001/18/EC^1^ Art. 16(1). And on scientific grounds such as low risk, an application might dispense with part of the information for post-release handling Directive 2001/18/EC, Art. 13(2). In addition, for the environmental risk assessment, the information required “may vary depending on the type of the GMOs concerned” [Directive 2001/18/EC, Annex II (B)]. In fact, individual applications for release shall not be required to present information “where it is not relevant or necessary for the purposes of risk assessment in the context of a specific notification” (Directive 2001/18/EC, Annex III, as amended by Commission Directive 2018/350). In short, risk assessment can and should be tailored to the type of GMO in question and GEOs are definitely a special type of GMOs (cf. similar arguments by Bratlie et al., 2019 and Eriksson, 2018b).

Third, the Directive does not prescribe specific scientific methods of risk assessment (as is normal for a legal act of this category), i.e., specific qualitative vs. quantitative methods, which varieties to compare with, in the lab or in the field, null hypotheses, sample sizes, specific values for statistical significance etc. It simply lists different risks that have to be assessed and maintains that studies have to conform to usual standards (Directive 2001/18/EC, Annex II (C), as amended by Commission Directive 2018/350). Consequently, authorities could draw more strongly than they do now on different kinds of readily available evidence including theoretical considerations and evidence from published scientific literature on similar types of GMOs. In particular, it should in most cases suffice to “refer to data or results from notifications previously submitted by other notifiers” cf. Directive 2001/18/EC, Art. 6(3) and of course to evidence from “releases of the same GMOs […] outside the Community” cf. Directive 2001/18/EC, Art. 13(3); note that in the case of GEOs we will soon have abundant information. The type of scientific evidence (e.g., from a study conducted on the whole organisms vs. from theoretical considerations that estimate risks) and the level of detail required in response to each subset of considerations should be allowed “to vary according to the nature and the scale of the proposed release” (cf. Directive 2001/18/EC, Annex III). Note that this is not meant as a cut on rigor of risk assessment but only as a methodological change within the bounds of scientific method choice. Why should from a scientific standpoint a single locally confined field trial be more representative for the effects of a specific trait than knowledge from its agricultural use across decades? In the case of GEOs the traits are often already known from decades of cultivation in conventionally bred varieties. In the most simple terms: Systematic reviews and evidence maps based on known risks have to suffice when no scientific hypothesis is available against which empirical testing can be done, which is the case for many GEOs (as discussed above).

Of course, the flexibility extant in the GMO Directive is a breadless argument in some respects. On the one hand, even if a soft administrative change along these lines was accomplished, applications for placing on the market of GMOs could still get stuck in the committee voting procedures. Consequently, the timespan from notification until committee voting might decrease, but after voting there wouldn't necessarily be more successful authorizations than today—unless member states bring into better alignment their committee votes or other incentives are given (as e.g., the instatement of a “GMO opt-in mechanism” proposed by Eriksson et al., 2018). On the other hand, regulatory agencies are not free to act on the Directive directly but they have to take into account how the Directive has been transposed in the member states. In addition, while the GMO Directive (2001/18/EC) is the centerpiece of GMO legislation, other regulations are in place that narrow the flexibilities discussed above. Especially the regulations for GMO food and feed (discussion of Commission Implementing Regulation EU No 503/2013^7^ below) do lay out the process in much more detail and in a more restrictive manner that leaves less freedom to treat GEOs any differently than transgenic GMOs. In practice, most GMOs apply for market approval as food and feed. Still, there are field trials and releases for non-food&feed purposes (e.g., industrial enzymes or raw materials for the production of biopolymers, biofuels, paper, starch etc.; EFSA, 2009) that do not in principle fall under that regulatory regime and could therefore profit from more flexibility, as regulated according to part B and part C of Directive 2001/18/EC respectively. And the Commission Implementing Regulation (EU) No 503/2013 is not itself completely devoid of flexibility, e.g., its Art. 5(2) concedes that an application may have to fulfill less requirements if this can be justified for the GMO in question.

The take home message is that if member states and their regulatory agencies are willing, slightly defusing legal uncertainties for GEOs is already possible by adjusting procedures and communicating transparently, even before tackling legislation. While the aspect of flexible implementation should not be underestimated, especially since it is the first thing that will happen, the fundamental problems of GMO law cannot be solved by such means.

### Amend Commission Implementing Regulation (EU) 503/2013

Applications for authorization for placing on the market of GMOs as food and feed are regulated among others by the Regulation (EC) No 1829/2003 on Genetically Modified Food and Feed which is implemented by the Commission Implementing Regulation (EU) No 503/2013 on Applications for Authorisation of Genetically Modified Food and Feed. While the Regulation 1829/2003 and other regulations leave a lot of flexibility for regulatory agencies to shape the process of assessment, the Commission Implementing Regulation (EU) No 503/2013 lays down concrete criteria and prescribes scientific methods to be applied by regulatory agencies. The two most time consuming and costly scientific requirements of the assessment process are:

1. The environmental risk assessment, which has to be carried out with field trials on a minimum of 8 sites that have to be representative for the GMOs receiving environment and often cover multiple years [cf. Commission Implementing Regulation EU No 503/2013, Annex II, II. Sci. Req. 1.3.2.1(b)].
2. The mandatory toxicology assessment by 90-day feeding studies on rodents (cf. Commission Implementing Regulation EU No 503/2013, Annex II, II. Sci. Req. 1.4.4.1).

Both these assessment procedures have been scientifically supervised and evaluated. The procedure of environmental risk assessment has been criticized repeatedly for leading to results that are not comparable, as no common test protocol is implemented (e.g., Hilbeck and Otto, 2015; Priesnitz et al., 2016; Fernández Ríos et al., 2018). Moreover, it has recently been suggested to adapt the methods of risk assessment for new types of GMOs (Duensing et al., 2018). The current regime of 90-days feeding studies for toxicology assessment has been criticized, as “the performance of rat feeding trials with whole food/feed for the risk assessment of a GM plant would not result in additional information pointing at possible health risks” as compared to less expensive studies and biochemical characterization (G-TwYST, 2018; cf. similar results in GRACE, 2018). In other words, both these tests are lengthy and expensive but do not seem suited to reveal new risks that cannot also be investigated by other means. These results call for a change in assessment procedures for all GMOs.

Regarding GEOs in particular, the addition of a caveat explicitly exempting them from such studies (90-day feeding studies and extended field trials), provided that the alterations introduced are deemed non-hazardous based on theoretical considerations and currently available scientific evidence, would constitute a decisive improvement. With any such update to Commission Implementing Regulation (EU) No 503/2013, the European Commission can significantly lower regulatory hurdles for agricultural innovators. On the other hand, GEOs would still be subject to all other obligations of GMO law and many applications that are geared toward more agricultural sustainability, with small alterations that improve existing varieties, will not become economically attractive unless exempted from all obligations of GMO law.

### Update Annexes II, III, VI, VII of GMO Directive 2001/18/EC

An additional option for lowering the regulatory burden for innovators bringing GEOs to market is an update to technical progress to Annexes II, III, VI, and VII of the GMO Directive, which is provided for by statute with recourse to a Committee Procedure that has to be initiated by the European Commission (Directive 2001/18/EC, Art. 27 i.c.w. Art. 30(2), referring to Committee Procedure of Decision 1999/468/EC). An explicit exemption for GEOs for some of the obligations laid down in these Annexes might have an effect if followed by a diligent implementation in the concomitant regulations. However, as a single measure, upgrading the annexes is not adequate to mitigating the uncertainties for GEOs, as the Directive already leaves relative freedom to deal with different types of GMOs and individual cases (discussion above). By contrast, the fundamental problems lie in the main body of the GMO Directive (2001/18/EC) and its Annex IB, as these define the scope of the law. This part, however, cannot be tackled by a Committee Procedure but requires an ordinary legislative procedure.

### Amend, Supplement or Replace GMO Law (Primarily Directive 2001/18/EC)

Being more transparent and flexible regarding regulatory procedures (section Make Use of Flexibility Within Current Legal Framework) and lowering the costs of applications for market approval of GEOs (section Amend Commission Implementing Regulation) is not enough. The most significant uncertainties stem from foreseeable problems with enforcement, particularly when taking into account global trade (discussion in section The ECJ Judgment and its Ramifications). Regarding GEOs specifically, it has to be somehow ensured that organisms that cannot be distinguished from organisms bred with conventional techniques, which currently do not fall under the obligations of the GMO Directive, are regulated equally–or at least in a pragmatic manner similarly, also taking into account economic feasibility. Solving this issue is the centerpiece to finding a solution to the new uncertainties in European GMO regulation.

The following options could accomplish this task, for example (in any combination):

- Amend the GMO Directive (2001/18/EC), for example by (A) revising the wording of the GMO definition in Art. 2(2) in order to exclude organisms that could also have been obtained by conventional breeding techniques or/and (B) updating the mutagenesis exemption in Art. 3 i.c.w. Annex IB to additionally exclude GEOs (cf. Dutch Proposal, 2017) or/and (C) clarifying what constitutes a “long safety record” in the sense of Rec. 17.
- Supplement the GMO law by a new act that specifically applies to GEOs, at least among others. This is maybe politically more feasible and efficient than opening up the entrenched debates on adequate regulation for GMOs in general. It has also been suggested to transfer competencies back to member states for specific cases (cf. the idea of an “opt-in Directive” by Eriksson et al., 2018; slightly outdated scenarios for regulation of NBTs in Purnhagen et al., 2018a). The drawback of additional acts is that the core of GMO law will still have to be revised at a later stage, as new technological developments will again put pressure on old GMO law, such as epigenetic modifications or gene drives (gene drives are not GEOs in the sense of this article but transgenic organisms and the scientific community is very much aware that they present high risks unless very specific conditions are met, cf. Akbari et al., 2015;Brown, 2017).
- In terms of complete replacement of GMO law, there are plenty of options. An obvious choice would be to develop an outcome-based regulation (e.g., novel trait), as this is a means to ensure that the law automatically keeps up with technological progress (cf. Huang et al., 2016). Opponents of the current horizontal legislation of GMO release might propose replacement by sectorial regulation, as this allows a more customized treatment of e.g., plant varieties with higher starch content vs. e.g., future medical applications that may require a permit for release. A new tiered approach has recently been proposed by the Norwegian Biotechnology Advisory Board (Bratlie et al., 2019).

This article does not provide further discussion and evaluation of such options. There is no shortage of ideas for renewed legislation and it is far too early to hypothesize on which options might find political majorities more easily. In order to solve the issue of GEOs, the broad goals of amending, supplementing or replacing legislation are clear: (1) Updating the regulatory framework in a way that specifically addresses GEOs, including issues of regulatory enforcement. (2) Making the regulatory framework more flexible to future developments in green biotechnology and allowing fast and predicable incorporation of recent scientific evidence. (3) Finding a balance between precautionary regulation and allowing sustainable innovation. European legislation is a lengthy process and that is why the involved actors have to start acting soon.

Although this is speculative, in several decades new technologies will be developed that might be shaped by the current regulatory hurdles for GMOs. For example, techniques of conventional mutagenesis, which are currently exempted from all obligations for GMOs, might be developed further and maybe employed differently, in order to allow much faster and efficient breeding, e.g., in combination with future enhanced molecular markers. And in the long run, it might become possible to circumvent all aspects of the current regulatory regime by developing breeding techniques that do not change the “genetic material” of an organism (see remarks in Table 1). Such future breeding techniques could draw on e.g., epigenetic modifications (cf. Thakore et al., 2016), CRISPR-interference (cf. Dominguez et al., 2016), mRNA interference or proteome modifications. Either way, as new breeding methods develop over the next decades, the gap between the scope of the current regulatory framework and technical possibilities will widen and put additional pressure on a complete overhaul of European GMO law.

## Conclusion

Companies and research institutions that employ new breeding techniques are confronted with considerable legal uncertainties after the recent ECJ judgment. While in principle uncertainties tied to the process of application for authorization of GMO release can be addressed by procedural changes on a lower level, problems of enforcement with organisms that are indistinguishable from the result of conventional breeding techniques cannot be solved without an amendment of European GMO legislation. There are various options for legal change that all share the common necessity of treating organisms that are indistinguishable from non-GMOs equally if they are devoid of known additional risks—that is, to exclude them from most or all obligations of GMO regulation as well. This is by no means a statement for less rigor, as organisms with novel traits that are associated with risks should still be assessed and regulated thoroughly.

Any such solution however requires the constructive involvement of European institutions and member states. The roads forward presented in this article are thus mere possibilities in an optimistic scenario that presupposes political willingness to act. This may not be realistic in the current political situation. However, if the problems in GMO law are just ignored a state of crisis will ensue: Regulatory agencies will struggle to enforce GMO-regulation, international trade relations will be affected, European agriculture loses an opportunity for sustainable innovation and jobs in research and development will be relocated elsewhere.

## Author Contributions

The author confirms being the sole contributor of this work and has approved it for publication.

### Conflict of Interest Statement

The author declares that the research was conducted in the absence of any commercial or financial relationships that could be construed as a potential conflict of interest.

## Footnotes

^1^*Directive 2001/18/EC of the European Parliament and of the Council of 12 March 2001 on the Deliberate Release into the Environment of Genetically Modified Organisms and Repealing Council Directive 90/220/EEC*. 106, 1–39. Available online at: http://eur-lex.europa.eu/eli/dir/2001/18/oj (accessed March 1, 2018).

^2^*Regulation (EC) No 1946/2003 of the European Parliament and of the Council of 15 July 2003 on Transboundary Movements of Genetically Modified Organisms (Text with EEA relevance)*. 287, 1–10. Available online at: http://data.europa.eu/eli/reg/2003/1946/oj (accessed March 1, 2018).

^3^*Regulation (EC) No 1829/2003 of the European Parliament and of the Council of 22 September 2003 on Genetically Modified Food And Feed (Text with EEA relevance)*. 268, 1–23. Available online at: http://data.europa.eu/eli/reg/2003/1829/oj (accessed March 1, 2018).

^4^*Regulation (EC) No 1830/2003 of the European Parliament and of the Council of 22 September 2003 Concerning the Traceability and Labeling of Genetically Modified Organisms and the Traceability of Food and Feed Products Produced from Genetically Modified Organisms and Amending Directive 2001/18/EC*. 268, 24–28. Available online at: http://data.europa.eu/eli/reg/2003/1830/oj (accessed March 1, 2018).

^5^Commission Directive EU No 2018/350 of 8 March 2018 amending Directive 2001/18/EC of the European Parliament and of the Council as Regards the Environmental Risk Assessment of Genetically Modified Organisms. 67, 30-45. Available online at: http://data.europa.eu/eli/dir/2018/350/oj (accessed September 12, 2018).

^6^*Directive 2009/41/EC of the European Parliament and of the Council of 6 May 2009 on the Contained Use of Genetically Modified Micro-organisms*. 125, 75–97. Available online at: http://data.europa.eu/eli/dir/2009/41/oj (accessed March 1, 2018).

^7^*Commission Implementing Regulation EU No 503/2013 of 3 April 2013 on Applications for Authorisation Of Genetically Modified Food and Feed in Accordance With Regulation (EC) No 1829/2003 of the European Parliament and of the Council and Amending Commission Regulations (EC) No 641/2004 and (EC) No 1981/2006 (Text with EEA relevance)*. 157, 1–48. Available online at: http://data.europa.eu/eli/reg_impl/2013/503/oj (accessed March 1, 2018).
